# Mixed acinar-neuroendocrine carcinoma of the pancreas with positive for microsatellite instability: a case report and review of the literature

**DOI:** 10.1186/s40792-023-01709-5

**Published:** 2023-06-30

**Authors:** Kenji Yoshino, Yosuke Kasai, Manabu Kurosawa, Atsushi Itami, Kyoichi Takaori

**Affiliations:** 1 Department of Surgery, Nagahama City Hospital, 313, Oinui-cho, Shiga 526-8580 Nagahama, Japan; 2 Department of Surgery, Nishi-Kobe Medical Center, 5-7-1 Koji-dai, Nishi-ku, Hyogo 651-2273 Kobe, Japan; 3 Department of Surgery, Graduate School of Medicine, Kyoto University, 54 Kawahara-cho, Shogoin, Sakyo-ku, Kyoto, 606-8507 Kyoto Japan; 4 Department of Pathology, Nagahama City Hospital, Nagahama, Japan

**Keywords:** Mixed acinar-neuroendocrine carcinoma, Microsatellite instability-high tumors, Programmed cell death-1 monoclonal antibody

## Abstract

**Background:**

Mixed acinar-neuroendocrine carcinoma (MANEC) of the pancreas is a rare tumor. We report a case of successful surgical resection of expansively growing MANEC of the pancreas with microsatellite instability (MSI)-high.

**Case presentation:**

The patient was an asymptomatic 65-year-old male. A computed tomography (CT) scan for a follow-up after treatment of pneumonia incidentally revealed a hypoenhancing 12-cm expansively growing tumor in the pancreatic body. An endoscopic ultrasound-guided fine-needle aspiration of the tumor suggested the diagnosis of MANEC. We performed distal pancreatectomy with combined resection of the spleen, left adrenal gland, transverse colon, small bowel, and stomach. The intraoperative findings showed that the tumor was capsular and was in contact with the SMA, SMV, and CA; however, obvious infiltration of these vessels was not observed..Pathological findings indicated MANEC with MSI-high. Among mismatch repair (MMR) gene proteins, PMS2 was lost and MLH1, MSH2, and MSH6 were retained. The tumor recurred 5 months after surgery. The patient was treated with gemcitabine plus nab-paclitaxel followed by pembrolizumab, which did not show objective response.

**Discussion:**

This is the first report investigating MSI and MMR in MANEC. Standard chemotherapy has not been established for MANEC. Detection of MSI-high is essential since PD-1 monoclonal antibodies for MSI-high cases might be one of the good treatment options. Herein, we discuss the various cytomorphologic and clinical features of MANEC and present a brief review of the literatures.

**Conclusions:**

The accumulation of data from additional cases is necessary to further evaluate this type of carcinoma and provide a standardized optimal therapy for MANEC.

## Introduction

A mixed acinar-neuroendocrine carcinoma (MANEC) is a rare pancreatic neoplasm, with less than fifty reported cases [1]. MANEC is a variant of acinar cell carcinoma (ACC) exhibiting neuroendocrine differentiation only immunohistochemically, and is distinguished from mixed-neuroendocrine-nonendocrine neoplasm (MiNEN) which is a mixture of acinar and neuroendocrine tumors confirmed by solely morphological features. The key diagnostic feature of MANEC is that MANEC expresses both neuroendocrine antigens (eg, synaptophysin and chromogranin) and pancreatic exocrine antigens (eg, trypsin and lipase). The behavior of MANEC may be similar to that of acinar cell carcinoma (ACC) [2], and surgical resection is the first choice, if the tumor is localized and resectable [1, 3]. However, with the limited number of reported cases of MANEC, its appropriate treatment modalities and overall prognosis remain unclear.

Recently, pembrolizumab, an anti-programmed cell death-1 (PD-1) monoclonal antibody, has been used in malignant solid tumors with microsatellite instability (MSI)-high. However, there have been no reports investigating MSI in MANEC. Herein, we present a case who underwent a successful surgical resection of an expansively growing MANEC of the pancreas with MSI-high and brief literature review.

## Case presentation

**Fig. 1:**
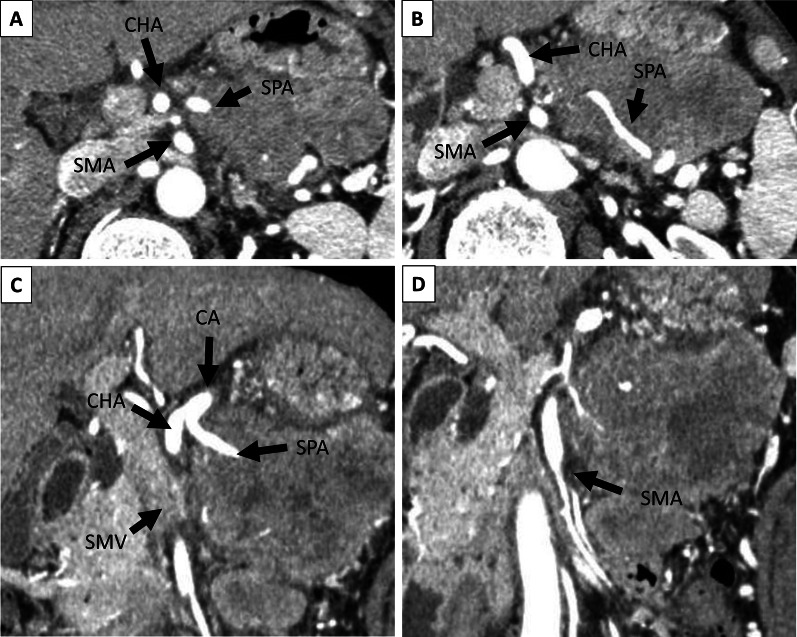
Preoperative contrast-enhanced computed tomography. The tumor was close to the celiac artery (CA), superior mesenteric artery (SMA) and vein (SMV), and common hepatic artery (CHA, **A**–**D**) The splenic artery was patent despite circumferential involvement by the tumor (**A**–**C**)

**Fig. 2:**
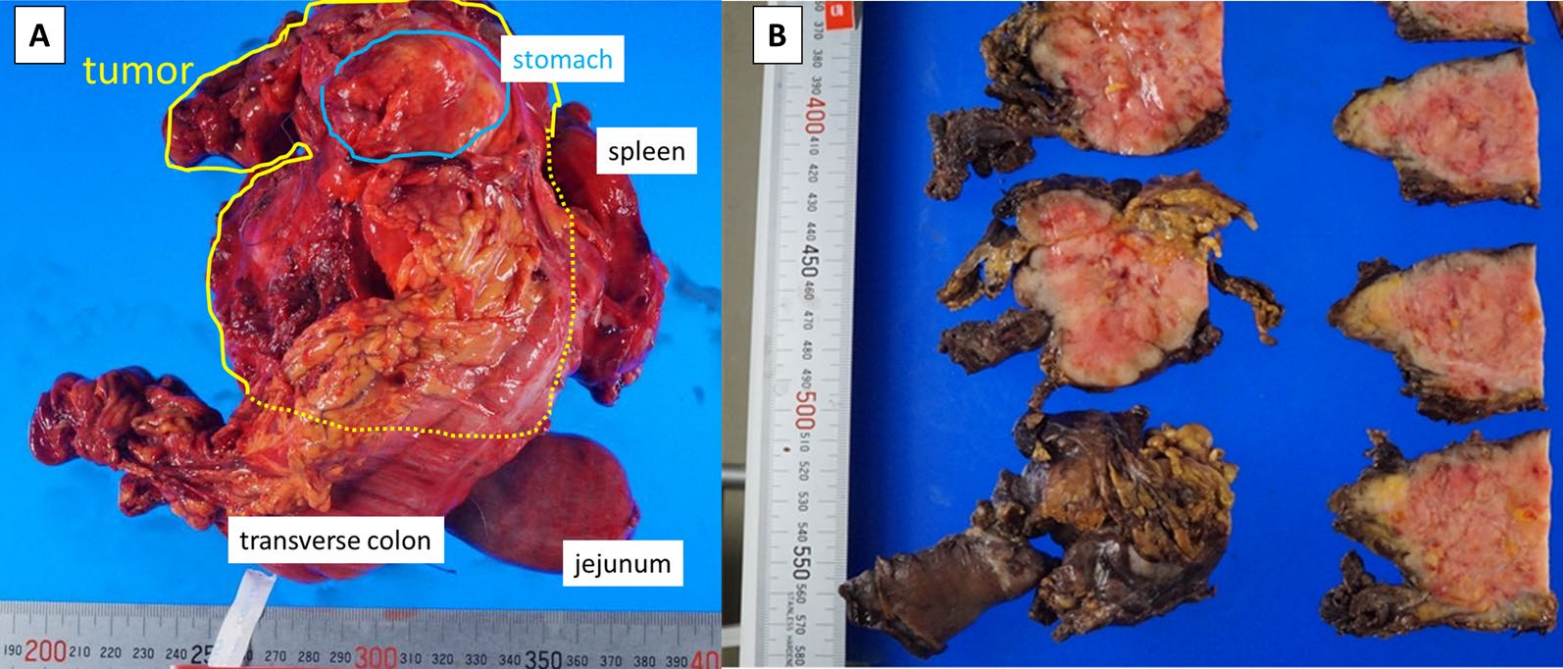
Macroscopic view of the resected specimen. Distal pancreatectomy with combined resection of the spleen, left adrenal gland, transverse colon, small bowel, and stomach (**A**). The tumor was encapsulated (**B**)

**Fig. 3:**
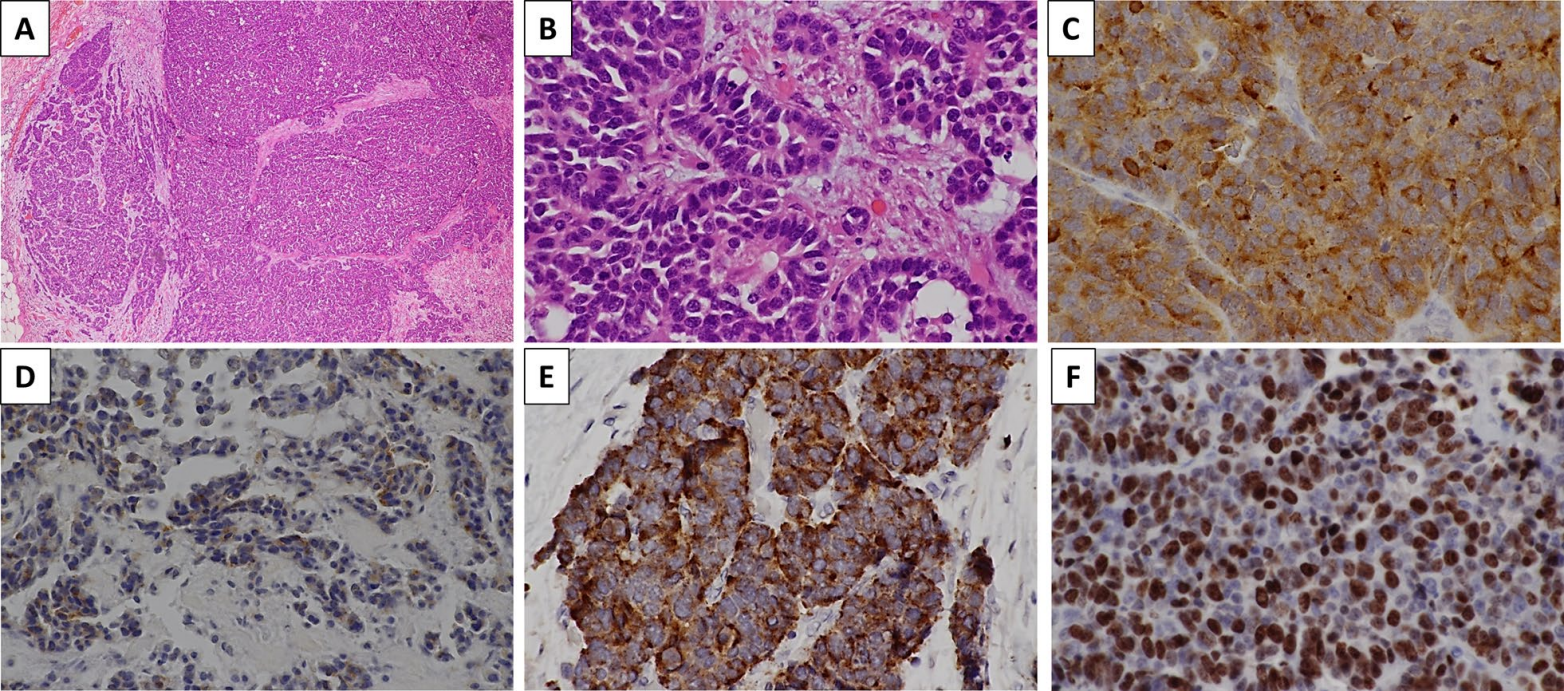
Histopathological findings. Hematoxylin and eosin staining at a magnification of **A** × 40 and **B** × 400; **C** BCL-10 staining, magnification, × 400; **D** trypsin staining, magnification, × 400; **E** chromogranin A staining, magnification, × 400; **F** MIB-1 staining, magnification, × 400). **A**, **B** Hematoxylin and eosin staining revealed an acinar growth of tumor cells with round nuclei and eosinophilic vesicles. Immunohistochemistry revealed positive BCL-10 (**C**), trypsin (**D**), and chromogranin A staining (**E**). The MIB-1 as Ki-67 expression was 80% positive (**F**)

**Fig. 4:**
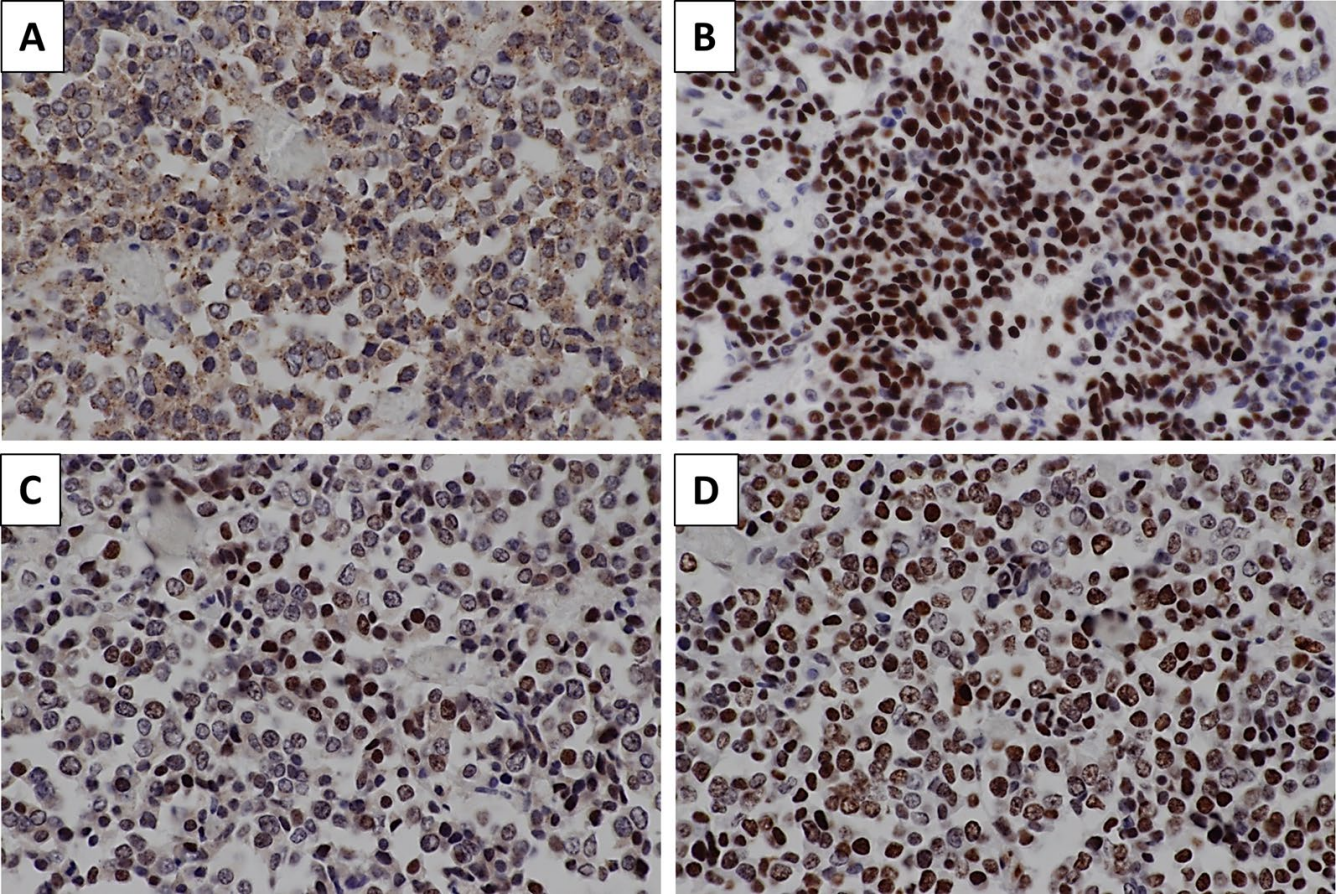
Immunohistochemistry for mismatch repair gene proteins revealed negative PMS2 (**A**) and positive MLH1 (**B**), MSH2 (**C**), and MSH6 staining (**D**)

**Fig. 5:**
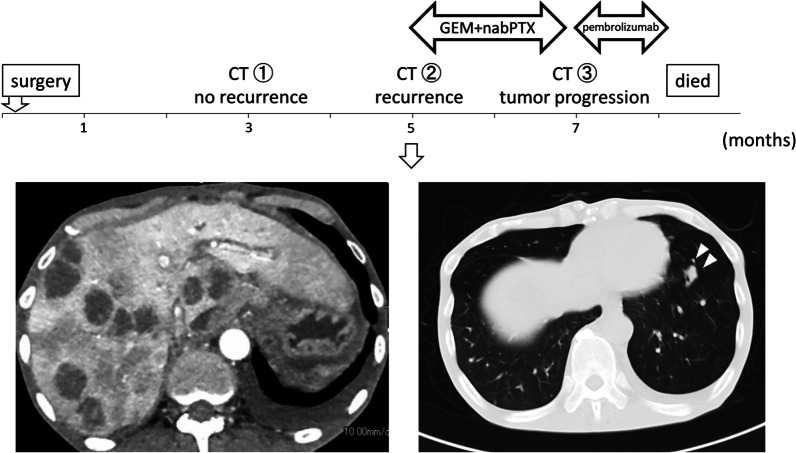
Summary of the treatments. Five months after surgery, a CT scan revealed the presence of multiple liver, lung, and lymph node metastases and peritoneal dissemination. Chemotherapy with gemcitabine (GEM) plus nab-paclitaxel (nabPTX) was administered. Subsequently, pembrolizumab was administered as the second-line treatment, however, did not show a good response

The patient was an asymptomatic 65-year-old man with a recent history of pneumonia. A follow-up computed tomography (CT) scan incidentally revealed a large tumor on his left upper abdomen. The patient did not have a reported family history of pancreatic cancer. Laboratory examinations showed elevated serum C-reactive protein (CRP) levels (2.76 mg/dl). His serum albumin and hemoglobin levels slightly decreased to 3.9 g/dl (normal range 4.1–5.1 g/dl) and 13.5 g/dl (normal range 13.7–16.8), respectively. His serum transaminase, pancreatic enzymes (amylase and lipase), lactate dehydrogenase (LDH), alkaline phosphatase (ALP), total bilirubin, blood glucose, and HbA1c levels were within normal limits. His serum elastase-1 level elevated to 3250 (normal range 0–300), while his carcinoembryonic antigen (CEA), cancer antigen 19–9 (CA19-9), duke pancreatic monoclonal antigen type 2 (DUPAN-2), and s-pancreas antigen-1 (Span-1) levels were within their normal ranges. A contrast-enhanced CT revealed a hypoenhancing 12-cm tumor in the pancreas body and tail, suggesting a primary pancreatic cancer. The tumor was close to the celiac artery (CA), superior mesenteric artery (SMA) and vein (SMV), and common hepatic artery (CHA, Fig. 1A–D). Notably, the splenic artery was patent despite circumferential involvement by the tumor (Fig. 1 A–C), which indicated the expansive-growth pattern of the tumor. The magnetic resonance imaging (MRI) showed that the tumor had a low intensity on the T1-weighted imaging, a high intensity on the T2-weighted imaging, and a marked restricted diffusion on the diffusion-weighted imaging. Neither CT nor MRI scans indicated any distant metastases. The patient underwent an endoscopic ultrasound-guided fine-needle aspiration (EUS-FNA) of the mass. This revealed the presence of tumor cells with round nuclei and eosinophilic to amphophilic granular cytoplasm. An immunohistochemical examinations showed that the tumor was positive for BCL-10, trypsin, chymotrypsin, chromogranin A (80% positive), and synaptophysin (20% positive). We diagnosed the tumor as a pancreatic ACC or MANEC. The patient underwent a complete surgical resection by distal pancreatectomy with combined resection of the spleen, left adrenal gland, transverse colon, small bowel, and stomach (Fig. 2). The intraoperative findings showed that the tumor was capsular and was in contact with the SMA, SMV, and CA; however, obvious infiltration of these vessels was not observed. The pathological examination indicated an MANEC with positivity for BCL-10, trypsin, chymotrypsin, and chromogranin A (Fig. 3A–E). The MIB-1 as per the Ki-67 expression was 80% positive in the immunochemical staining (UICC TNM classification 8^th^ edition: pT3, pN2, pMX StageIII) (Fig. 3F). The tumor tissue DNA was analyzed for genomic abnormalities, and the resected specimen indicated that the tumor was MSI-high and negative for RAS/BRAF mutation. Immunohistochemically, The mismatch repair (MMR) gene protein PMS2 was lost and MLH1, MSH2, and MSH6 were retained (Fig. 4). The patient did not receive any adjuvant therapy because of fatigue and loss of appetite. The progress after the surgery is shown in Fig. 5. Three months after the surgery, there was no recurrence of the tumor. However, five months after surgery, a CT scan revealed multiple liver, lung, and lymph node metastases and peritoneal dissemination. Chemotherapy with gemcitabine plus nab-paclitaxel was administered as the first-line treatment. Due to the side effects (leukopenia and severe malaise) and poor efficacy (tumors enlarged < 20%), the patient stopped receiving the treatment after two courses. Considering MSI-high of the tumor, pembrolizumab was administered as the second-line treatment. Although the patient tolerated this regimen, a CT scan revealed the tumor´s progression. The patient's general condition gradually deteriorated, and he died 8 months following the surgery.

## Discussion

MANEC of the pancreas is extremely rare, and very little is known about its clinical and radiological features and pathogenesis. Therefore, it is difficult to differentiate MANEC from ACC or endocrine neoplasms on clinical and radiological evaluations. EUS-FNA is the most common technique used for the diagnosis of pancreatic neoplasms, including MANEC, and it also plays a crucial role in determining the treatment and triage. However, there are only a few reports on the effectiveness of perioperative chemotherapy for MANEC [4–7], and a standardized management protocol for a pancreatic MANEC has not yet been established. Hence, it has been agreed that generally surgery is the sole curative therapy for resectable MANEC [8, 9].

The list of reports describing cases of MANEC is shown in Table 1. [1, 2, 4–6, 9–34] MANEC is common in males, and the susceptible age is 50–70 years old. Despite the presence of endocrine components, most patients do not have specific hormonal symptoms. Moreover, there are no useful tumor markers related to MANEC. Hence, MANEC is usually diagnosed at advanced stages [median size of 7.9 cm with synchronous distant metastases being present in 40.5% (17/42) of the cases]. These findings are based only on small case series or case reports with very short follow-up periods (median 12 months), and they do not thoroughly discuss the treatment strategies and their effects.

**Table 1:** Literature review of the reported cases diagnosed with mixed acinar-neuroendocrine carcinoma

Author	Publication date	Age	Symptom	Sex	Size (cm)	Metastasis	Treatment	Regimen of chemotherapy	Follow-up	Recurrence
Ulich et al	Nov, 1982	30	Epigastric pain	F	9.3 × 8.6 × 5.8	None	PD		Alive (4 months)	–
Ichijima et al	Jan, 1985	6	–	F	8 × 5 × 4	None	Resection		Alive (13 years)	–
Hassan et al	Sep-Oct, 1993	50	Weight loss, constipation, abdominal pain	M	19 × 18	Liver, multiple	DP + splenectomy + colectomy		Deceased (10 months)	
Klimstra et al	Aug, 1994	81	Nausea, abdominal pain	M	3	None	Resection		Alive (3 months)	–
		70	Back pain	M	4 × 10	Liver, lungs, lymph nodes	Resection		Deceased (3 months)	
		64	Hematemesis	F	10	None	Bypass (inoperable)		Deceased (18 months)	–
		48	–	F	11	Liver	Resection		Alive (12 months)	
		79	Abdominal pain	F	10	None	Bypass chemoradiation	N/A	Alive (12 months)	–
Cho et al	Apr, 1996	52	Jaundice	F	6 × 6	None	PD + SMV resection		Alive (12 months)	–
Shimoike et al	Dec, 1997	28	-	M	3	Liver, multiple; vertebral bone	TAE, chemotherapy	STZ, 5-FU	Deceased (10 months)	
Frank et al	Aug, 1998	61	Uncharacteristic abdominal complaints	M	4.9 × 3.6 × 2.8	Liver, later peritoneal carcinomatosis, skin	PD_+_ left hemihepatectomy + chemotherapy	Octreotide, a-interferon	Deceased (3.5 years)	
Muramatsu et al	Sep, 2000	72	Appetite loss, weight loss	M	12 × 13	Liver, multiple	N/A		Deceased (3 months)	
Ogawa et al	Jun, 2000	50	-	M	3 × 2.5	None	PD		Alive (18 months)	–
Skacel et al	Sep, 2000	69	N/A	M	10	None	Resection		Deceased (20 months)	+
		75	N/A	M	5.5	None	Resection		Alive (10 months)	–
Mizuno et al	May, 2001	67	-	F	N/A	Liver, multiple; at autopsy: spleen, stomach, left ovary, para-aortic lymph nodes	Chemotherapy	Octreotide, diazoxide, 5-FU, doxorubicin	Deceased (7 years)	
Ohike et al (6 cases)	Jul, 2004	Mean 58.4 (49–65)	N/A	M:F (2:4)	8.2 (n = 2)	N/A	N/A		N/A	–
Imaoka et al	Oct, 2008	80	N/A	M	4	None	PD		N/A	–
Kyriazi et al	Apr, 2009	74	-	M	12 × 9 × 6	None	PD		Alive (3 months)	–
Chung et al	Nov, 2010	59	Watery diarrhea	F	8 × 2.5	None	DP + splenectomy		N/A	–
Kobayashi et al	Apr, 2010	75	-	M	7	None	DP		Alive (6 months)	–
Soubra et al	Mar, 2013	52	Epigastric pain, fatigue, jaundice	M	1.5 × 1.2 × 1.0	Liver, soft tissue	PD + chemotherapy	1st: cisplatin, camtothecin 2nd: gemcitabine, cisplatin	Alive (30 months)	
Lee et al	Feb, 2013	66	Mid-epigastric discomfort	M	3.1 × 2.8	Liver	Chemotherapy	Irinotecan, capecitabine, erlotinib, docetaxel	Deceased (21 months)	
Sullivan et al	April, 2013	75	Acute pancreatitis	M	0.6	Liver	Chemotherapy	N/A	N/A	
		51	-	M	1.6	None	DP		N/A	–
Kanemasa et al	Sep, 2013	63	Left flank pain	M	6	Liver	Chemotherapy	1st: S-1 2nd: gemcitabine	Deceased (18 months)	
Ogbonna et al	Nov, 2013	57	Epigastric pain radiating to the back	F	2.5	None	DP + splenectomy + chemoradiation	etoposide,carboplatin	N/A	–
Yu et al	Apr, 2013	80	Epigastric pain associated with dyspepsia and early satiety	M	14 × 9	Liver, kidney	Chemotherapy, palliative surgery	1st: carboplatin, etoposide 2nd: FOLFOX	Alive (36 months)	
		89	Poor appetite and weight loss	M	3.9 × 3.7	None	PD		Deceased (2.5 months)	–
		60	Abdominal pain, diarrhea	M	16 × 13	None	Colectomy, gastrectomy, pancreatectomy, chemotherapy	Cetuximab, irinotecan	Deceased (6 months)	
		74	Epigastric pain, weight loss	M	10 × 5.5	None	Chemoradiation + DP	neoadjuvant: 5-FU	Alive (17 months)	–
		59	Fatigue, poor appetite, intermittent jaundice	M	7.5 × 6.5	None	Whipple		Alive (7 months)	Local recurrence and liver (4 months)
Kumamoto et al	Apr, 2015	42	Back pain	M	3.1 × 8.0 × 2.9	Liver, multiple	DP + hepatectomy		Alive (48 months)	4 months
Liu et al	Aug, 2015	65	Jaundice, nagging epigastric pain, intermittent diarrhea	F	8 × 6	Liver, multiple	PD + partial hepatectomy		Alive (12 months)	
Sugimoto et al	Apr, 2017	48	Abdominal pain	M	7.7 × 5.9	None	Chemotherapy + PD	Neoadjuvant: FOLFIRINOX	Alive (21 months)	–
Takano et al	Oct, 2017	50	Epigastralgia and back pain	M	3	None	DP		Alive (1 year)	–
Hara et al	Dec, 2017	45	Severe anaemia	F	11	Liver, multiple; later, stomach	TACE followed by DP + splenectomy; later gastrectomy		Alive (7 years)	
Strait et al	Nov, 2018	33	Abdominal discomfort, back pain, early satiety, weight loss	M	3.6 × 2.9	Liver, multiple	Chemotherapy + PD	Neoadjuvant: FOLFIRINOX	Alive (10 months)	
		66	Abdominal pain	M	10	None	Chemotherapy	FOLFIRINOX, panitumumab	Alive (4 months)	–
Tang et al	Jun, 2019	52	Tenderness in the right upper quadrant	M	10.6	Liver, multiple	Chemoradiation	Etoposide, cisplatin	Deceased (5 months)	
Niiya et al	Oct, 2020	72	Upper abdominal pain	M	2.9	None	DP		N/A	–
Akki et al	Jul, 2021	60 s	Abdominal pain	M	2.2	None	PD		Alive (31 months)	liver
		60 s	-	M	4	None	DP		Alive (6 months)	-
our case		65	–	M	12	None	DP + splenectomy + left adrenal, transverse colon, small bowel resection, partial gastrectomy, chemotherapy	1st: gemcitabine, nab-paclitaxel 2nd: pembrolizumab	Deceased (8 months)	liver, lung, lymph node, peritoneal dissemination (5 months)

Concerning neoadjuvant therapy, the usefulness of FOLFIRINOX has only been described in two case reports [6, 35]. Yu et al. performed chemoradiation therapy with 5-fluorouracil; however, the effectiveness of the treatment was not evaluated [5].

The malignant potential of MANEC is considered to be less than that of pancreatic ductal adenocarcinoma. MANEC has the characteristics of expansive growth, a well-defined margin with a capsule, and a lack of or relatively mild vascular and bile duct encasement [36]. In our case, even though the tumor appeared to be in contact with major vessels such as CA and SMA on CT imaging, it did not actually infiltrate the vessels, and margin-negative curative resection was achieved through aggressive surgical procedure. Meanwhile, tumor recurrence was observed in approximately 50% of patients who underwent curative resection, indicating that micrometastases were already present, even in a clinically resectable MANEC. Therefore, adjuvant therapy may be considered to reduce recurrence risk and improve outcomes, even after a curative resection. However, there have been no reports with respect to adjuvant chemotherapy following surgery for MANEC. For unresectable or recurrent MANEC, there are only a few case reports of treatment with FOLFIRINOX, FOLFOX, and S-1 and their effectiveness is not clear. [4–7] Currently, the efficacy of anti-PD-1 monoclonal antibodies for MSI-high solid tumors has attracted considerable attentions. This is the first report investigating MSI in MANEC. In MiNEN, several reports have examined the presence of MSI. Sahnane et al. reported a MSI-high rate of 12.4% in gastroenteropancreatic neuroendocrine carcinoma and MiNEN tumors [37]. Additionally, Ishida et al. studied MiNEN tumors of the stomach and reported MSI- high rate of 7.7% [38]. The tumor was MSI-high and out of the four mismatch repair genes, only PMS2 was inactivated. In a previous report, PMS2 was required for the cisplatin-induced activation of p53, which is a member of the p53 family of transcription factors with proapoptotic activity in ovarian cancer [39]. Furthermore, Jia et al. reported that PMS2 expression was regulated post-translationally by Akt and was essential for the platinum-induced apoptosis in ovarian cancer [40]. Thus, the efficacy of chemotherapy may differ depending on the type of MMR deficiency. A PMS2 deficiency may be associated with the efficacy of chemotherapy. Although the recurrent tumors in our case did not show a good response to pembrolizumab despite the MSI-high status, pembrolizumab may be one of the good treatment options for MSI-high MANEC, given the lack of robust evidence of chemotherapies for MANEC. In our case, a mutation in the RAS/BRAF gene mutation was not identified. In previous reports, only two reports have investigated genetic mutation in MANEC [30, 34]. Therefore, further investigations are needed to expand our understanding of genetic mutations in MANEC.

## Conclusion

While surgical resection remains the first choice for the treatment of MANEC without distant metastasis, new modalities such as anti-PD-1 monoclonal antibodies may be considered for advanced MANEC with MSI-high. The accumulation of more data from additional cases is necessary to further evaluate this type of carcinoma and provide a standardized optimal therapy for MANEC.

## Data Availability

The data that support the findings of this manuscript are available from the corresponding author, Kenji Yoshino, upon reasonable request.

## Abbreviations

ACC: Acinar cell carcinoma
ALP: Alkaline phosphatase
CA: Celiac artery
CA19-9: Cancer antigen 19–9
CEA: Carcinoembryonic antigen
CHA: Common hepatic artery
CRP: C-reactive protein
CT: Computed tomography
DUPAN-2: Duke pancreatic monoclonal antigen type 2
EUS-FNA: Endoscopic ultrasound-guided fine-needle aspiration
LDH: Lactate dehydrogenase
MANEC: Mixed acinar-neuroendocrine carcinoma
MiNEN: Mixed-neuroendocrine-nonendocrine neoplasm
MMR: Mismatch repair
MRI: Magnetic resonance imaging
MSI: Microsatellite instability
PD-1: Anti-programmed cell death-1
SMA: Superior mesenteric artery
SMV: Superior mesenteric vein
Span-1: S-pancreas antigen-1
